# pMPES: A Modular Peptide Expression System for the Delivery of Antimicrobial Peptides to the Site of Gastrointestinal Infections Using Probiotics

**DOI:** 10.3390/ph9040060

**Published:** 2016-10-02

**Authors:** Kathryn Geldart, Brittany Forkus, Evelyn McChesney, Madeline McCue, Yiannis N. Kaznessis

**Affiliations:** 1 Department of Chemical Engineering and Materials Science, University of Minnesota, 421 Washington Ave SE, Minneapolis, MN 55455, USA; gelda002@umn.edu (K.G.); forku001@umn.edu (B.F.); 2 Breck School, 123 Ottawa Ave N, Golden Valley, MN 55422, USA; mcchesney.evelyn@gmail.com (E.M.); maddy.mccue@gmail.com (M.M.)

**Keywords:** antimicrobial peptides, microcin V, heterologous production, secretion, *E. coli* Nissle 1917, antimicrobial probiotics

## Abstract

Antimicrobial peptides are a promising alternative to traditional antibiotics, but their utility is limited by high production costs and poor bioavailability profiles. Bacterial production and delivery of antimicrobial peptides (AMPs) directly at the site of infection may offer a path for effective therapeutic application. In this study, we have developed a vector that can be used for the production and secretion of seven antimicrobial peptides from both *Escherichia coli* MC1061 F’ and probiotic *E. coli* Nissle 1917. The vector pMPES (Modular Peptide Expression System) employs the Microcin V (MccV) secretion system and a powerful synthetic promoter to drive AMP production. Herein, we demonstrate the capacity of pMPES to produce inhibitory levels of MccV, Microcin L (MccL), Microcin N (McnN), Enterocin A (EntA), Enterocin P (EntP), Hiracin JM79 (HirJM79) and Enterocin B (EntB). To our knowledge, this is the first demonstration of such a broadly-applicable secretion system for AMP production. This type of modular expression system could expedite the development of sorely needed antimicrobial technologies.

## 1. Introduction

Antibiotic treatments have defined a bright era in human history, during which human life expectancy has increased and quality of life has improved substantially. However, this era may be coming to an end because of the emergence of bacterial strains that are resistant to even the most potent of antibiotics. Consequently, an urgent need exists to develop new, alternative antimicrobial strategies.

Herein, we present tools for a new antibiotic technology that targets pathogenic bacteria. We modified probiotic bacteria to express and deliver antimicrobial peptides in the gastrointestinal (GI) tract of hosts.

Antimicrobial Peptides (AMPs) are naturally produced by living organisms as a first line of defense against invading bacteria. A plethora of AMPs have well-characterized, strong and specific activity against pathogenic bacteria. AMPs have thus been posited as promising alternatives to traditional small-molecule antibiotics [1,2,3,4].

In practice, however, AMP therapeutic utility is limited by high production costs and poor bioavailability profiles. Often, adverse toxicity profiles prohibit systemic administration, and oral administration often results in rapid peptide degradation. This degradation limits oral delivery of functional AMPs to the gastrointestinal (GI) tract, where many infections originate.

We hypothesize that we can overcome the AMP delivery challenge for the treatment of GI tract infections by engineering probiotic bacteria to produce and secrete AMPs at the site of intestinal infections.

Probiotics are bacteria that are generally regarded as safe for consumption, are often naturally beneficial to the consuming host, and can survive passage through the GI tract. The most common bacteria considered probiotics are lactic acid bacteria (LAB), including the genera *Lactobacillus, Lactococcus*, and *Bifidobacterium* [5]. These organisms are not virulent and possess the fitness capacity necessary for intestinal colonization and survival. Moreover, numerous probiotic organisms naturally produce endogenous AMPs called bacteriocins [6,7,8]. With demonstrated beneficial properties, probiotic effects on mammalian health have been linked with the ability to produce bacteriocins. Probiotics have thus been proposed as a means for maintaining and improving gut health.

Probiotic organisms have also been considered as potential vehicles for drug delivery in the GI tract [9]. *L. lactis* is a model LAB for heterologous protein secretion [8], and has potential applications for the treatment of human Crohn’s disease by the production of interleukin-10 in the GI tract [10]. The probiotic *Escherichia coli* strain Nissle 1917 has also been studied extensively and tested as a solution to inflammatory bowel disease [11].

Several expression systems have been developed in probiotics, including the NIsin Controlled gene Expression (NICE) system [12], and expression systems that are inducible under various conditions such as phage attack, temperature, or pH shift [13].

In our group, *Lactococcus lactis*, *Lactobacillus* spp. and probiotic *Escherichia coli* Nissle 1917 [1,14,15,16], have been engineered to produce specific AMPs. In our previous work, we demonstrated the expression of AMPs and their secretion in inhibitory amounts against various pathogens, including *Enterococcus faecalis, Enterococcus faecium* [1,14], *Escherichia coli* [15], and *Salmonella enterica* [16].

While these systems are useful for the expression of certain peptides targeting a specific pathogen of interest, a production and secretion system that can be used for a wide array of peptides from a single delivery organism may constitute a powerful, flexible platform for designing therapeutic antimicrobial probiotics. Herein, we present the design and testing of such a system for the potential delivery of multiple AMPs in the GI tract of hosts.

If the goal is delivery of inhibitory levels of AMPs at the site of an intestinal infection, then secretion is one of the primary obstacles to overcome. Many AMPs are secreted by dedicated ATP-binding cassette (ABC) transport systems [17,18,19,20,21]. In these cases, the AMP is expressed with an N-terminal signal peptide recognized by the transport machinery. Upon exit from the cell, the tag is cleaved from the peptide, and the mature, active AMP is released [22].

Previous studies have demonstrated that in some cases, AMPs can be secreted from a heterologous host’s secretion machinery by replacing the native signal peptide with the signal peptide associated with the host’s own machinery. For example, the *Enterococcus*-derived AMPs Enterocin A and Hiracin JM79 (HirJM79) have been successfully secreted by the *Lactococcus lactis* and *Lactobacillus* native general secretion machinery, using the *Lactococcus*-derived Usp45 signal peptide [23,24,25].

In another study, it was shown that Carnobacteriocin B2 could be secreted through the *Carnobacterium piscicola* general secretion pathway using the Divergicin A signal peptide [26]. Yet another study showed that Divergicin A fused to the Leucocin A (LeuA) leader peptide could be secreted using the Leucocin A, Lactococcin A (LcnA) and Microcin V (MccV) secretion machineries [22].

Though this method of signal peptide exchange shows promise, successful production from the systems studied thus far is generally unpredictable and is highly dependent on the particular AMP, transport system and signal peptide components tested. Additionally, to our knowledge, no studies have ever tested the secretion of more than two or three AMPs from a given transport system.

In this study, we have created an AMP expression vector, pMPES (Modular Peptide Expression System), which employs a well-characterized *E. coli* AMP secretion system to produce and secrete a variety of AMPs. We start by discussing the development of this vector. We then investigate its ability to produce inhibitory amounts of seven different AMPs, three derived from *E. coli* and four derived from *Enterococcus*. Lastly, we show that we are able to use this vector to simultaneously produce combinations of AMPs targeting both Gram-positive and Gram-negative bacteria. Collectively, the system described herein helps build the foundation for a generalized AMP production system.

## 2. Results

### 2.1. Development of the Vector

The purpose of this study was to create a bacterial vector for the production and secretion of a variety of AMPs from a single probiotic organism. We remodeled a MccV production plasmid, pHK22, in order to create a vector that contained the entire MccV secretion machinery, as well as a strong DNA promoter system and an AMP cloning site.

**Figure 1:**
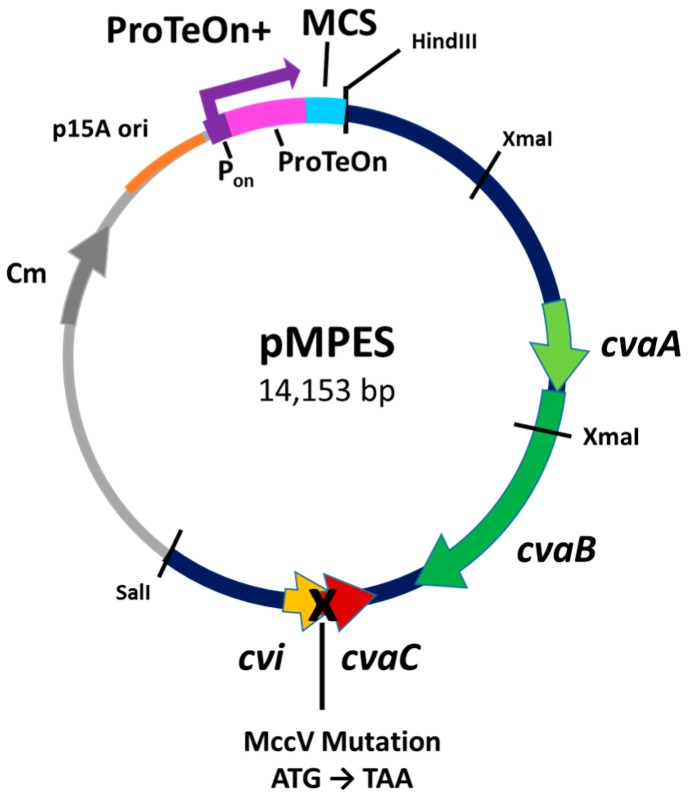
Diagram of pMPES (Modular Peptide Expression System). ProTeOn+: synthetic DNA promoter; P_on_: promoter region; ProTeOn: activator protein; *cvaA/cvaB*: MccV section machinery; *cvaC*: MccV peptide (native); *cvi*: MccV immunity protein; Cm: Chloramphenicol resistance; MCS: Multiple Cloning Site.

We refer to this final vector as pMPES, for Modular Peptide Expression System. Figure 1 shows a diagram of this vector.

The MccV secretion system was selected for this application because it is compatible with *E. coli*, because it is among the most well-characterized AMP secretion systems and because it recognizes a glycine-glycine type signal peptide, a common class of signal peptide used by many AMPs [22].

The MccV secretion pathway relies on the ABC transporter CvaB and the accessory protein CvaA [18,27,28]. The outer membrane protein, TolC, is encoded in the *E. coli* chromosome and is also required for transport [27,29,30].

Vector pHK22 contains a 9.1-kb fragment originally isolated from the native MccV production plasmid, pColV-K30. This region, located between the HindIII and SalI restriction enzyme cut sites shown in Figure 1, was previously found to contain all of the necessary components for native MccV production, immunity and secretion [18]. The sequence of this region is reported under GenBank Accession Number X57524.1 [27].

For the development of pMPES, we made three primary modifications to pHK22: (1) we mutated the MccV structural gene to remove native AMP activity; (2) we added a well-characterized synthetic DNA promoter; (3) we added a multiple cloning site and a terminator site to facilitate insertion and removal of AMPs.

The mutation of the native *mccV* gene was essential for testing of pMPES, since MccV production would interfere with the characterization of other peptides. This mutation was introduced into the start codon of the native *mccV* gene, *cvaC*, to encode a stop codon as shown in Figure 1. This new vector is referred to as pHK22Δ. Mutation of the stop codon was selected over complete removal of *mccV* in order to minimize the risk of disrupting any unknown secretory components. To verify that MccV or any potentially active truncated peptides were no longer being produced, *E. coli* MC1061 F’ containing pHK22Δ was tested against the indicator strain, *E. coli* DH5α, and shown to have no activity (Figure S1).

Next, a strong DNA promoter was incorporated into the vector to enable high-level protein expression. We have previously developed a synthetic DNA promoter for *E. coli,* which relies on a synthetic hybrid activator protein, ProTeOn [31]. ProTeOn was constructed by physically linking the reverse tetracycline repressor protein to the activating domain of the *Vibrio fischeri* transcription factor, LuxR. ProTeOn makes strong contacts within the engineered DNA promoter site (P_on_), which contains optimally-spaced tetracycline and LuxR operator binding regions. This system recruits RNA polymerase and strengthens the holoenzyme-DNA interactions to up-regulate gene expression.

ProTeOn has been further modified in this study to include a positive feedback loop by inserting the gene encoding ProTeOn downstream of Pon. By using this feedback loop, we are able to amplify promoter expression and obtain high levels of the proteins of interest compared to the original ProTeOn promoter. Herein, we will refer to this new expression construct as ProTeOn+.

A multiple cloning site (MCS) containing the five restriction sites SacI, ApaI, AvrII, NotI and PciI and a rho-independent terminator sequence was then inserted downstream of ProTeOn+.

### 2.2. Activity Tests

In this study, we evaluated the production of seven different peptides from the MccV secretion machinery; MccV, Microcin L (MccL), Microcin N (McnN), Enterocin A (EntA), HirJM79, Enterocin P (EntP) and Enterocin B (EntB). These peptides were selected to represent a wide range in percent sequence identity to MccV and its production system. We note that these tests were not intended to be comprehensive, but only indicative of combinatorial possibilities.

MccV, MccL and McnN are natively produced by *E. coli* while the remaining peptides are produced by *Enterococcus*, a Gram-positive genus of bacteria [17,19,23,32,33]. Like MccV, the studied enterocins are considered class II bacteriocins, which generally lack major post-translational modifications and are commonly secreted by ABC-transporters using N-terminal secretion tags [34].

With the exception of EntP and HirJM79, all of the peptides tested herein are naturally encoded with glycine-glycine leader peptides [17,19,33,35]. EntP and HirJM79 are believed to be secreted via the general Sec-type secretion pathways of their native producers [3,36].

Table 1 shows the percent identity and similarity of the signal peptides, the mature peptides and the two primary secretion genes associated with MccL, McnN and EntA compared to MccV. The EntB, HirJM79 and EntP transporter genes are not compared because they are either unknown as in the case of EntB or they belong to a different class of transporters [3,36,37]. Identity and similarity values were calculated using the EMBOSS Needle (European Molecular Biology Open Software Suite version 6.6.0.0, Needleman-Wunsch global alignment application) global sequence alignment program with default parameters [38].

Based on the alignment results, one can see that the signal peptides of all three AMPs share significant similarity to MccV’s signal peptide. These similarities are further discussed below. Importantly, the transporters for MccL and MccV are nearly identical despite the differences in the mature peptides.

**Figure 2:**
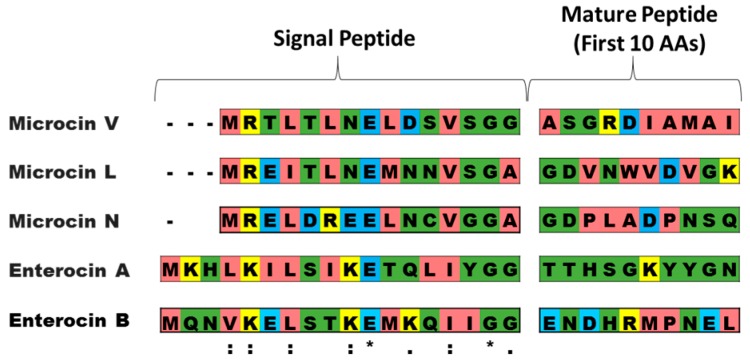
Alignment of signal peptides and first ten amino acids of class II peptides selected for initial activity tests. Pink residues indicate small and hydrophobic amino acids (AVFPMILW); yellow indicates basic amino acids (RK); blue indicates acidic amino acids (DE); and green indicates hydroxyl/sulfhydryl/amine/G amino acids (STYHCNGQ). * means fully conserved residues; : means high conservation (scoring >0.5 in the Gonnet PAM250 matrix); . means low conservation (scoring <0.5).

Figure 2 shows an alignment of the secretion signal peptides and the first ten amino acids of the mature peptides. The first ten amino acids were included in the alignment because it has been previously hypothesized that the amino acids adjacent to the signal peptide may significantly impact secretion and processing [39]. The multiple sequence alignment program Clustal Omega was used for alignment results [40]. Amino acid conservation scores are based on the Gonnet PAM250 substitution matrix. Residues with a score >0.5 are considered highly conserved in similarity, and those with scores <0.5 are considered to have low conservation.

In addition to testing the capacity of the MccV secretion system with seven different AMPs, another objective of this study was to compare the effect of different signal peptides on active peptide secretion. Consequently, in addition to testing peptide production using the MccV signal peptide (denoted Vsp), we also tested the production of MccL, McnN and EntA using their own, native signal peptides. For EntA, the impact of fusing the MccL signal peptide to EntA (denoted LspA) was also tested because of the high level of similarity between MccV and MccL secretion tags and transporters.

Ultimately, the combination of distinct AMPs and of separate secretion signal tags resulted in eleven systems, listed in Table 2.

In order to reduce the toxicity of the constructs to the producer strain, the immunity genes MccV, MccL and McnN were included in constructs encoding these peptides. Immunity genes were not included for HirJM79, EntP or EntB because these peptides were previously found to be inactive against the producer strain, *E. coli* MC1061 F’.

**Figure 3:**
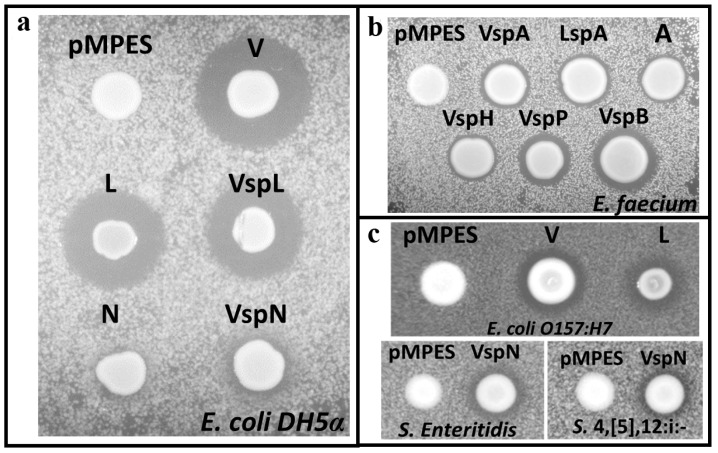
(**a**) Activity of MccV, MccL and McnN from *E. coli* MC1061F’ using the pMPES production system. *E. coli* DH5α was used as the indicator strain on this agar plate; (**b**) Production and secretion of EntA, HirJM79, EntP and EntB from *E. coli* MC1061F’ using pMPES. *E. faecium* 8E9 was used as the indicator strain on this agar plate; (**c**) Inhibition of *E. coli* O157:H7 by *E. coli* MC1061F’ pMPES:V and L (top) and *Salmonella enterica* serovar Enteritidis and *Salmonella enterica* serovar 4,[5],12:i:- by pMPES:VspN. In the figure, Vsp and Lsp indicate the use of the MccV or MccL signal peptide rather than naturally-encoded signal peptide.

Agar diffusion assays with appropriate indicator strains were used to screen for peptide activity from the different constructs. Figure 3 shows the agar diffusion assay results of *E. coli* Mc1061 F’ containing the nine AMP constructs listed in Table 2, as well as the empty control, pMPES.

For these tests, 3 μL of overnight producer strain culture were spotted on agar containing the indicator strain and then incubated overnight. The white spots are the producer strain, and the dark regions surrounding the producer are zones of inhibition.

Inhibition is likely the result of expressed and secreted AMPs. We acknowledge that in this study, despite our efforts, no peptides were isolated and quantitatively measured as direct proof that inhibition was due to their production and secretion. This may be due to the small culture sizes used (40 mL), which may not produce sufficient peptide amounts for detection using canonical protein isolation methods. It may be useful to scale up culture sizes to one liter or more and to test alternative concentrating, sodium dodecyl sulfate polyacrylamide gel electrophoresis (SDS-PAGE) and purification methods for each of the tested systems, but this was beyond the scope of this study.

Nevertheless, all negative controls, which are included for all studies for activity comparison, suggest that antimicrobial activity is indeed the result of secreted AMPs. This conclusion that AMPs are expressed and secreted is further supported by the fact that in all cases, activity was observed only against the expected indicator strain. For example, MccL constructs were inactive against the Gram-positive *E. faecium*, whereas EntA constructs were inactive against the Gram-negative *E. coli* DH5α.

For these assays, MccV, MccL and McnN were tested against *E. coli* DH5α (Figure 3a) because it has previously been used as the indicator strain for MccV and McnN [13,23]. EntA, HirJM79, EntP and EntB were tested against *E. faecium* 8E9 (Figure 3b) because these peptides were previously shown to have activity against this strain [1,14]. Note, *E. faecium* 8E9 is a multidrug-resistant pathogenic isolate.

MccL and MccV producers were also tested against foodborne pathogen *E. coli* O157:H7 (Figure 3c, top), and McnN producers were tested against foodborne pathogens *Salmonella enterica* serovar Enteritidis and *Salmonella enterica* serovar 4,[5],12:i:- (Figure 3c, bottom) [41,42].

In Figure 3a, one can see clear zones of inhibition around *E. coli* MC1061 F’, pMPES:V, L, VspL and VspN. These results strongly suggest that all of these strains are producing and secreting inhibitory levels of their respective peptides.

Similarly, results in Figure 3b suggest the production and secretion of EntA, HirJM79, EntP and EntB using all signal peptides tested. The activity depicted in Figure 3c demonstrates that the V, L and VspN constructs are potent enough to exhibit activity against pathogenic *E. coli* and *Salmonella* strains.

In order to further validate the hypothesis that the peptides were in fact being secreted using the MccV secretion machinery, negative controls were made for VspA, L and N. For these controls, *cvaA* and *cvaB* were removed from the vector to abolish MccV secretion by digesting pMPES with XmaI (see Figure 1) then relegating the digestion. Using agar diffusion tests, no activity was detected from any of these negative controls.

In the future, we aim to use this type of AMP production system to deliver peptides using probiotic bacteria. We therefore sought to test pMPES’s compatibility with probiotic *E. coli* Nissle 1917. The pMPES:V, L and VspA constructs were transformed into *E. coli* Nissle 1917 and tested using agar diffusion assays (Figure S2). Definitive activity could be detected from all three constructs compared to the negative control, suggesting that the pMPES expression and secretion systems are compatible with Nissle. Figure S3 shows an additional agar diffusion assay, the results of which are consistent with those shown in Figure S2. Note that, unlike *E. coli* MC1061 F’, *E. coli* Nissle 1917 naturally produces AMPs, Microcin H47 and Microcin M, which could account for the activity observed in the negative controls against *E. coli* DH5α [43]. In Figure S3, it appears that VspN shows a more defined halo than pMPES, implying, but not definitively determining activity.

### 2.3. Simultaneous Expression of Multiple AMPs

One of the primary benefits of a flexible secretion system for AMP production is the potential to simultaneously produce multiple peptides from a single construct. To verify this potential with the pMPES secretion system, we assembled a construct containing VspA and L. Note that this construct employs a different Ribosomal Binding Site (RBS) upstream of the peptides. We therefore refer to this backbone as pMPESb. The sequences of the pMPES and pMPESb RBS’s are provided in Table S1.

**Figure 4:**
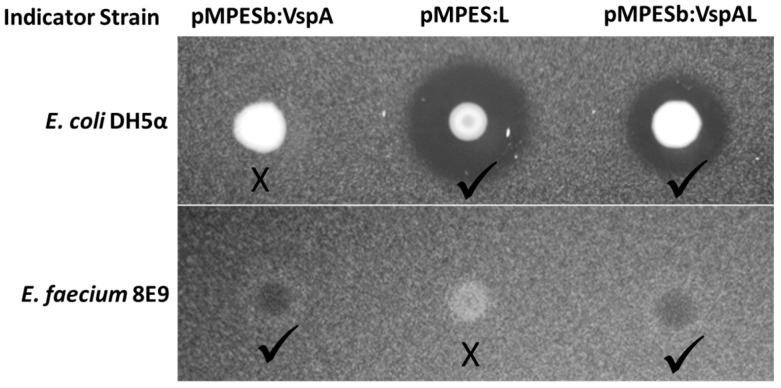
Simultaneous production of EntA and MccL from *E. coli* MC1061 F’ pMPESb:VspAL and comparison to single AMP systems. Note, pMPESb has a different Ribosomal Binding Site (RBS) and exhibits reduced peptide activity.

Figure 4 shows agar diffusion assays of *E. coli* MC1061 F’ pMPESb:VspAL on both *E. coli* DH5α and *E. faecium* 8E9. This figure suggests simultaneous secretion of both EntA and MccL. We recognize that the zones of inhibition presented here are less prominent than those observed using pMPES. Based on comparisons of the VspA supernatant from pMPES versus pMPESb, we believe the alternative RBS drastically reduces AMP expression compared to pMPES. This statement is supported by translation rates estimated using the Ribosomal Binding Site calculator [44]. Nevertheless, we present these results as a proof-of concept, albeit it a rather weak one, for multiple peptide production.

### 2.4. Supernatant Activity Assays

**Figure 5:**
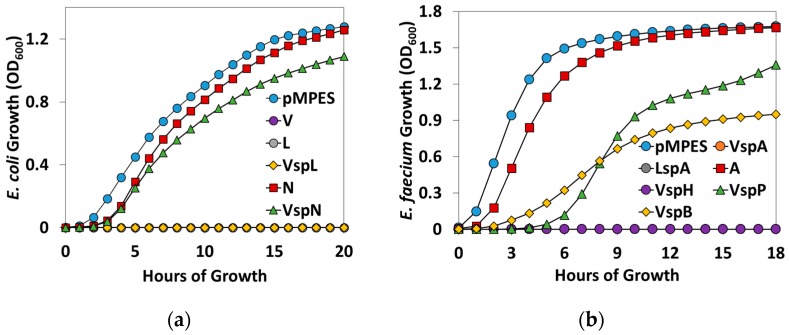
Liquid inhibition assays of the pMPES construct supernatants against: (**a**) *E. coli* DH5α and (**b**) *E. faecium* 8E9. Cultures are grown in 75% supernatant, 25% rich medium. Note, the V, L, VspL, VspA, LspA and VspH curves remain at OD_600_ = 0 on their respective graphs.

To quantitatively compare the activities of the different constructs, we performed liquid supernatant inhibition assays. Figure 5 shows the growth curves of the two indicator strains in the presence of 75% supernatant from the constructs discussed above. These growth curves are averaged over three biological replicates. On the left, *E. coli* DH5α growth curves are shown in the presence of supernatant from *E. coli* MC1061 F’ pMPES (negative control), the MccV, MccL and McnN constructs. On the right, *E. faecium* 8E9 growth is shown in the presence of supernatant from pMPES, VspA, LspA, A, VspH, VspP and VspB constructs.

Note that V, L, VspL, VspA, LspA and VspH curves remain at OD_600_ = 0 on their respective graphs. No regrowth was observed in any of these cultures after 48 h, indicating complete killing of the indicator strains, which were originally inoculated at ~5 × 10^3^ CFU/mL. In this study, an OD_600_ between ~0.05 and 1 is approximately linearly correlated with CFU/mL.

To further quantify the effectiveness of the constructs, the activity of the supernatants containing the AMPs was evaluated based on liquid growth assays. We stress that without the specific activities of the individual peptides, peptide production cannot be quantitatively compared across the different AMPs. Unfortunately, we were unable to isolate the peptides in any of the supernatants after multiple SDS-PAGE and HPLC attempts (see Materials and Methods). Additionally, it is important to consider peptide stability, particularly when comparing supernatant activity in which the calculated activity depends on peptide accumulation over several hours. Nevertheless the tests herein are potentially useful in comparing the efficacy of different signal peptides for a given AMP. In the case of the enterocins, which were tested against a pathogenic strain, these tests provide insight into which constructs may be the most potent for future application in probiotics.

Table 3 reports the inhibitory activities of the supernatants in terms of Bacteriocin Units (BUs) [23]. One BU is defined as the reciprocal of the highest dilution of supernatant required to reduce the growth of the indicator strain, with *p* < 0.05 compared to growth with pMPES supernatant. For these studies, supernatant was diluted in 2x dilutions from 0.75 down to 0.0059 (1.3–170 BUs). Reported values are the average of three biological replicates. Error represents the standard deviation of these replicates. Note, a higher number of supernatant BUs indicates greater potency against the indicator strain.

Table 3 also contains previously-reported minimum inhibitory concentrations (MICs) of the peptides in nM. We acknowledge however that these values were obtained by a variety of methods using different indicator strains than those used here. These values only are provided to give some idea of the potential activity of the different peptides, but should not be directly compared to this study.

Inhibition with p < 0.05 was observed for all supernatants tested in 75% supernatant. Interestingly, pMPES:L was more potent than pMPES:VspL, implying that the naturally-encoded signal peptide was more effective than Vsp for this particular AMP. This is in contrast to what was observed with EntA and McnN (see the growth curves and Figure 3a).

It should be noted that in multiple trials, VspA slightly out-performed LspA, but the difference is not observable in the BU calculations provided here.

## 3. Discussion

Delivery of AMPs to intestinal sites of infection poses a major challenge in their application as therapeutic antimicrobial agents. By engineering probiotic bacteria to produce AMPs at the site of infection in the GI tract, we can enable the delivery of otherwise unusable peptides, reduce the amount of peptide required and eliminate the need for protein purification. Furthermore, cocktails of peptides may result in synergistically higher activities and plausibly reduce the occurrence of resistance emergence.

To date, most AMP production systems have been created or tested with at most two or three distinct peptides. Ultimately, we aim to develop a library of probiotics that can be rapidly modified to produce a wide array of AMPs targeting different pathogens of interest. A more general AMP expression and secretion system would facilitate the development and testing of these new AMP-based probiotics. In this study, we have developed an AMP-production vector, pMPES, that can be used to produce a variety of AMPs from a single delivery organism.

Secretion has proven to be a major hurdle and area of interest in studies of heterologous AMP production. In this study, we evaluated the flexibility of the MccV secretion machinery contained in pMPES for heterologous AMP production by testing the production of seven different AMPs ranging in similarity to MccV. Note that for this study, AMP production was measured indirectly in the form of AMP activity. As shown in the Results section, all seven peptides tested with the pMPES vector could be detected at some level using inhibition assays. We acknowledge however that future improvements will likely be necessary to achieve levels of pathogen inhibition required for therapeutic applications.

The use of alternative signal peptides is a common approach for improving heterologous secretion [22,48,49,50]. We thus compared the activities of MccL, McnN and EntA constructs employing their naturally-encoded signal peptides versus the MccV signal peptide. Interestingly, while Vsp improved EntA and McnN construct activity, it actually hindered MccL activity compared to the naturally-encoded signal peptides. These results imply that the Vsp signal peptide may be more reliable for dissimilar AMPs, while the naturally-encoded signal peptide may be more effective when significant homology exists between the peptide/secretion machinery of the AMP and MccV.

Numerous other studies have also examined heterologous secretion of AMPs from other secretion pathways. Among the most interesting was a study in which Divergicin A (DivA) was shown to be secreted from the Leucocin A Lactococcin A and MccV secretion machineries using their respective signal peptides [22]. Additionally, this study also showed that the LeuA signal peptide could drive DivA secretion from the MccV machinery, but not the LcnA machinery. Furthermore, it was shown that MccV could be secreted from the LeuA machinery using the LeuA signal peptide. These peptides and secretion machineries are highly diverse, making this study particularly insightful.

In another study, chimeras of MccV and Microcin H47 (MccH47) were shown to be secreted from the MccH47 secretion machinery using either the MccV or MccH47 signal peptides [51]. It was believed that this capability was due to the similarity in transport proteins. The ABC transporter of MccH47 is 89% identical (93% similar) to that of MccV, and the accessory proteins are 42% identical (62% similar). Similarly, the high level of homology between the signal peptides and primary secretion components of MccL and MccV may explain why the Lsp signal peptide appears to have been efficiently recognized by the MccV machinery. Interestingly however, very little homology exists between MccL and MccV in the first 50 amino acids following the signal peptides, a region previously hypothesized to be of potential importance to secretion efficiency [39]. Collectively, these studies imply that while homology can in some cases predict successful AMP secretion, lack of homology does not necessarily result in secretion failure.

We are still in a period of attempts largely based on trial-and-error. However, we can imagine that this increasing body of knowledge can begin to facilitate the rational design of AMP expression and secretion systems. In the future, we can explore additional secretion systems, AMPs and signal peptides. Additionally, high-throughput screening methods can be used to select peptides from mutagenesis libraries exhibiting increased activity. Such a method would also in theory select for mutants with increased secretion efficiency.

In the future, in addition to improving peptide secretion, we will also explore possible improvements in gene expression and protein translation to increase overall peptide production. As mentioned in Section 2.3, RBS optimization offers a promising next step, since we have previously observed that even small differences in the RBS can drastically alter observed activity. Additionally, though we have previously observed ProTeOn and ProTeOn+ promoters to be highly active in *E. coli*, there may still be some room for improvement. It may also be of use to explore alternative origins of replication and to remove unnecessary components in the 9.1-kb MccV production region to reduce the burden of the vector on the host and to improve plasmid stability.

The ability to modularly express AMPs from probiotics would drastically facilitate the development of AMP-based probiotics. Though much work remains to be done, the study herein provides a foundation for a general AMP expression system. With this foundation, studies may be launched on the efficacy, safety and ADME (adsorption, distribution, metabolism and excretion) properties of antimicrobial probiotics, as well as on questions related to the use of genetically-modified live biotherapeutic bacteria, including environmental release and DNA transfer.

## 4. Materials and Methods

### 4.1. Bacterial Strains and Plasmids

The bacterial strains and plasmids used in this study are listed in Table 4.

#### Bacteria Growth Conditions

*E. coli* and *Salmonella* strains were grown with agitation in Luria-Bertani (LB) broth at 37 °C. *E. faecium* was grown in Brain Heart Infusion (BHI) medium at 37 °C in static conditions. When appropriate, chloramphenicol was added to the medium at a concentration of 20 µg/mL for *E. coli*.

### 4.2. Construction of Plasmids

Column or gel purification of digested vector backbone was performed using the Qiagen QIAquick PCR Purification Kit (Qiagen, Hilden, Germany) or the Gel Extraction Kit (Qiagen), respectively. Column purification of all PCR-amplified inserts and insert digests was done using the Qiagen Minelute DNA purification kit (Qiagen). Aside from colony PCRs, all PCRs used NEB Phusion^®^ High-Fidelity DNA Polymerase (New England Biolabs Inc., Ipswich, MA, USA). Colony PCRs used Promega GoTaq^®^ Green Master Mix (Promega, Madison, WI, USA). Ligations were done using NEB T4 DNA ligase, and assemblies were done using NEBuilder^®^ HiFi DNA Assembly Master Mix (NEB). Electrocompetent *E. coli* MC1061 F’ from Lucigen (Lucigen, Middleton, WI, USA) were used in all transformations unless otherwise stated. Electrocompetent *E. coli* Nissle 1917 was made as previously described [52]. Nissle was electroporated in a 2-mm cuvette under standard conditions [53]. All restriction enzymes were purchased from NEB. All procedures were done according to the manufacturer’s protocol unless stated otherwise. Primer and DNA fragment sequences are provided in Table S1. Successful transformants for pMPES, pMPESb and all AMP vectors were first screened using colony PCR with primers pHK22 HindIII Seq F and pHK22 HindIII Seq R and were then verified with Sanger sequencing.

pHK22Δ *cvaC* mutation: The start codon of the *cvaC* gene (sequence provided below) in pHK22 was mutated from ATG to TAA to create pHK22Δ. Note that the gene is encoded on the reverse strand, such that the mutation was CAT to TTA. The mutation was introduced in a piecewise fashion via PCR. First, two fragments were amplified from pHK22, so as to introduce the mutation at their overlap. The fragments were then fused and reinserted into pHK22. Fragment A (~1.9 kb), which sits between the BssHII restriction site and the *cvaC* start codon, was generated by PCR using forward primer SDML and reverse primer SDM R. The primer-introduced mutation is underlined in Table S1. Fragment B (~0.5 kb), which sits between the *cvaC* start codon and the BglII restriction site, was generated by PCR using forward primer SDM F and reverse primer SDML R. Purified Fragments A and B were then fused using a PCR reaction with primers SDML F and SDML R to give Fragment C (~2.4 kb). Purified Fragment C and pHK22 were then digested with BssHII and BglII. Fragment C digest was column purified, and pHK22 was gel purified to isolate the ~10.5 kb fragment generated. The pHK22 backbone and Fragment C were then ligated using T4 DNA ligase to form pHK22∆, and the resulting ligation was transformed into electrocompetent *E. coli* MC1061 F’. Successful transformants were first screened using colony PCR with primers SDM_seq_F and SDM_seq_R. Correct mutation was then verified with Sanger sequencing.

pMPES ProTeOn+ insertion: The ProTeOn+ DNA fragment was first amplified using Proteon_Assembler_F and Proteon_Assembler_R to give the ProTeOn Plus fragment provided in Table S1. Purified HindIII-digested pHK22∆ and ProTeOn Plus insert were then fused using the NEB HiFi assembly kit.

AMP Insertion into pMPES:MccL, Vsp:MccL, McnN, Vsp:McnN, EntA, Vsp:EntA, Lsp:EntA, Vsp:HirJM79, Vsp:EntP and Vsp:EntB gblocks were ordered from Integrated DNA Technologies (IDT), and the MccV fragment was ordered from Geneart. The sequences of these DNA fragments are provided in Table S1. McnN, Vsp:McnN and Vsp:EntA were inserted directly into SacI-PacI digested pMPES using the NEB HiFi assembly kit. In contrast, MccL,Vsp:MccL and Lsp:EntA were first amplified using the forward primer AMP F, and the reverse primer AMP R. MccV was amplified using MccV_SacI_F and MccV_PacI_R. EntA was amplified using EntA_pHK22ΔP F/R. Vsp:HirJM79, Vsp:EntP and Vsp:EntB were amplified using pMPES transition_F/R. The resulting MccL, Vsp:MccL, Lsp:EntA and MccV inserts were then digested using SacI and PacI restriction enzymes, column purified, then ligated into SacI-PacI-digested pMPES using T4 DNA ligase. EntA, VspHirJM79, VspEntP and VspEntB PCR products were column purified then assembled into SacI-AvrII-digested pMPES:N.

Negative controls of MccV, MccL, VspL, McnN and VspA were generated by digesting the pMPES vectors with XmaI restriction enzyme to remove the essential MccV secretion genes *cvaA* and *cvaB* (see Figure 1). Vectors were then reclosed via ligation. Successful transformants were first screened using colony PCR with primers MccV SeC F SpeI and CvaB seq R and were then verified with Sanger sequencing using CvaB seq R.

pMPESb: The original purpose of creating pMPESb was to create a more modular multiple cloning site. However, we later found the RBS to be less effective, and thus, pMPESb was abandoned for the majority of this study. The gblock encoding EntA was made so as to include the pMPESb multiple cloning site, the sequence of which is provided below. The AMP-free pMPESb was thus generated by first assembling the EntA gblock into SacI-PacI-digested pMPES. The resulting vector was then digested with SacI to cleave out EntA and the immunity gene. The purified digestion was re-ligated with T4-DNA ligase to produce pMPESb.

AMP insertion into pMPESb: To create pMPES_VA_L, VA and L inserts were amplified from pMPES:VspA and pMPES:L using PBS VA_For/Rev and PBS L_For/R. The resulting VEntA fragment was then amplified using R_For and O_Rev, and the MccL fragment was amplified using O_For and YG_Rev. The resulting fragments were then assembled into SacI-AvrII-digested pMPESb using the NEB HiFi-Assembler. To create pMPESb:VspA, the Vsp:EntA gblock for pMPESb was assembled directly into SacI-digested pMPES.

### 4.3. Activity Assays

#### 4.3.1. Agar Diffusion Assays

Figure 3a,b and Figure S2: 0.6 µL of indicator strain overnight culture was mixed with 100 µL of BHI medium and spread onto BHI agar plates. Plates were allowed to dry, then 3 µL of overnight producer culture were spotted onto the plate and allowed to dry completely. Dry plates were then covered and incubated overnight at 37 °C for imaging the following day.

Figure 3c and Figure S1: Liquid BHI agar was inoculated with 0.1 µL of *E. coli or Salmonella* overnight culture per mL medium. The inoculated agar was then poured into a petri dish and allowed to solidify. A 0.5-µL overnight culture of each *E. coli* strain was then dropped onto the plate with the appropriate indicator strain and allowed to dry completely. Dry plates were then covered and incubated overnight at 37 °C for imaging the following day.

Figure S3: Holes were cut into BHI agar plates then filled with liquid BHI agar containing 1000× dilution of Nissle overnight cultures and allowed to solidify, then sealed with 30 μL sterile BHI agar. Plates were incubated overnight at 37° C, then liquid BHI agar was inoculated with 0.5 μL/mL overnight indicator culture and poured over the producer plate. Dry plates were then covered and incubated overnight at 37 °C for imaging the following day.

#### 4.3.2. Supernatant Activity Assays

Producer and indicator strains were grown from plates for 12 h in 3 mL LB. Supernatants were filtered using a 0.22-µm filter (EMD Millipore, Billerica, MA, USA). Overnight indicator strain cultures were diluted in the appropriate growth medium by 10^5^ to give ~5 × 10^3^ CFU/mL cells. Sixty-two-point-five microliters of the diluted cultures were then combined with 187.5 µL of the supernatant, which had been appropriately diluted with pMPES supernatant. The plate was then incubated for 20 h at 37 °C with fast orbital shaking in a Synergy H1 plate reader (BioTek, Winooski, VT, USA). One Bacteriocin Unit (BU) is defined as the reciprocal of the highest dilution of supernatant that resulted in an increase in the culture’s Time To Rise (TTR) compared to growth in the absence of AMPs. TTR values were calculated as the hours required to rise to ¼ of the maximum OD_600_ for a given indicator strain. TTRs were first determined for a minimum of three growth curves of the indicator strain in 75% pMPES supernatant. We considered this to be the baseline growth in the absence inhibition. *p*-values were then obtained from a left-tailed two-sample *t*-test comparing the TTR values for each supernatant concentration against the pMPES supernatant TTRs. BUs for each biological replicate were then reported as the reciprocal of the highest dilution of supernatant that resulted in an increase in the culture’s TTR compared to growth in the absence of AMPs with *p* < 0.05. Values reported in Table 3 are the averages and standard deviations of BUs of three biological replicates.

### 4.4. Peptide Isolation Attempts

Several attempts were made using SDS-PAGE with and without supernatant concentration steps to visualize and estimate absolute AMP production and secretion. NuPAGE Novex 4-12% Bis-Tris Protein Gels were used for all SDS-PAGE attempts (Life Technologies Carlsbad, CA, USA). Up to 40 mL of culture were concentrated for SDS-PAGE samples. However, we were unable to see bands using Coomassie-stained SDS-PAGE for any of the producing strains. We continuously encountered a blurred region in the gels at the molecular weight expected for the peptides (3.5–10 kDa). We suspect that even with sufficient quantities of peptides that this region may make visualization difficult. We were unable to resolve this issue using minimal media or desalting columns. Additionally, reverse-phase HPLC was performed on supernatants using a Dionex UltiMate 3000 UHPLC (Dionex, Sunnyvale, CA, USA) with an XBridge Peptide BEH C18 column (Waters Corp., Milford, MA, USA) but no distinct peaks could be linked to AMP activity due to background noise. In the future, we will attempt additional AMP isolation techniques, such as size exclusion column chromatography and mass spectrometry. We will also test alternative types of gels based on previous work in the literature.

## Figures and Tables

**Table 1:** Comparison of the MccL, McnN, EntA, EntB, HirJM79 and EntP components to MccV.

AMP	Signal Peptide	Mature Peptide	Transporter (CvaB)	Accessory (CvaA)
MccL	60%/86.7%	44.1%/51.0%	95.4%/97.3%	97.6%/99.3%
McnN	41.2%/47.1%	20.8%/26.4%	71.5%/85.0%	69.4%/84.5%
EntA	22.2%/50%	10.3%/14.0%	24.8%/43.4%	18.5%/37.0%
EntB	22.2%/50%	14.9%/18.8%	NA	NA
HirJM79	10.0%/16.7%	6.0%/6.9%	NA	NA
EntP	6.5%/19.4%	5.5%/11.0%	NA	NA

NA: Comparisons for EntB, HirJM79 and EntP transporters were not applicable.

**Table 2:** AMP constructs tested in this study.

Construct (pMPES:)	Signal Peptide	Mature Peptide	Immunity Gene Included
V	MccV	MccV	Yes
L	MccL	MccL	Yes
VspL	MccV	MccL	Yes
N	McnN	McnN	Yes
VspN	MccV	McnN	Yes
VspA	MccV	EntA	Yes
LspA	MccL	EntA	Yes
A	EntA	EntA	Yes
VspH	MccV	HirJM79	No
VspP	MccV	EntP	No
VspB	MccV	EntB	No

**Table 3:** Inhibitory activities of supernatants produced by *E. coli* MC1061 F’ with the pMPES AMP constructs.

Indicator	Construct (pMPES:)	Bacteriocin Units (BU) ^1^	Previously Reported MIC
*E. coli* DH5α	V	120.9 ± 86.2	0.1 nM (*E. coli* MH1) [45]
	L	>170.7	160 nM (*E. coli* ML-35p) [46]
	VL	142.2 ± 49.3	160 nM (*E. coli* ML-35p) [46]
	N	1.3 ± 0	150 nM (*S. Enteriditis*) [35]
	VspN	1.3 ± 0	150 nM (*S. Enteriditis*) [35]
*E. faecium* 8E9	VspA	7.2 ± 3.4	129 nM (*E. faecium* TUA 1344L) [47]
	LspA	7.2 ± 3.4	129 nM (*E. faecium* TUA 1344L) [47]
	A	1.3 ± 0	129 nM (*E. faecium* TUA 1344L) [47]
	VspH	21.3 ± 0	~0.2 nM (*E. faecium T136*) [23]
	VspP	2.7 ± 0	~0.4 nM (*E. faecium* T136) [3]
	VspB	2.7 ± 0	43.4 nM (*E. faecium* TUA 1344L ) [47]

^1^ One Bacteriocin Unit (BU) is defined as the reciprocal of the highest dilution of supernatant required to reduce the growth of the indicator strain; error represents the standard deviation of three biological replicates.

**Table 4:** Bacteria and plasmids used in this study.

**Strain**	**Description**	**Source**
*E. coli* MC1061 F'	Plasmid-free, recA+, non-amber suppressor strain	Lucigen
*E. coli* DH5α PRO	Derivative of *E. coli* DH5α; PN25/tetR, Placiq/laci, cloning host	Clontech
*E. faecium* 8E9	Ampicillin/vancomycin/linezolid resistant hospital isolate	UMN^1^ collection
*E. coli O157:H7* 472	Common pathogenic species	UMN^1^ collection
*S. Enteritidis* Mh91989	Chicken isolate; common pathogenic species	UMN^1^ collection
*S. 4,[5],12:i:-* Mh06225	Chicken isolate; common pathogenic species	UMN^1^ collection
**Plasmid**	**Description**	**Reference**
pHK22	pACYC184 derivative containing 9.1-kb MccV production fragment	[18]
pHK22Δ	pHK22 derivative with mutated *cvaC* gene	This study
pMPES	pHK22Δ derivative containing the ProTeOn+ promoter	This study
pMPES:V, L, VspL, N, VspN, VspA, LspA, A, VspH, VspP, VspB	See Table 2	This study
pMPESΔ	pMPES digested with XmaI to eliminate cvaA and cvaB expression	This study
pMPESΔ:V	pMPESΔ with V and MccV immunity gene	This study
pMPESΔ:L	pMPESΔ with L and MccL immunity gene	This study
pMPESΔ:VA	pMPESΔ with VspA and EntA immunity gene	This study
pMPESb	pMPES with alternative ribosomal binding site	This study
pMPESb:VspA	pMPESb with VspA	This study
pMPES_b:VspA_L	pMPESb with VspA and L with their immunity genes	This study

^1^University of Minnesota (UMN)

## Supplementary Materials

The following are available online at www.mdpi.com/1424-8247/9/4/60/s1, Figure S1: Abolishment of native MccV production by mutation of *cvaC*, Figure S2: MccV, MccL and Enterocin A production from pMPES in *E. coli* Nissle 1917, Figure S3: Alternative assay showing MccV, MccL, McnN and EntA production from pMPES:V, L, VspN, and VspA in *E. coli* Nissle 1917, Table S1: Primers and DNA fragments used in this study.
