# Supplementary material for: pMPES: A Modular Peptide Expression System for the Delivery of Antimicrobial Peptides to the Site of Gastrointestinal Infections Using Probiotics
Source: Pharmaceuticals (Basel). 2016 Oct 2;9(4):60. doi: 10.3390/ph9040060 (PMC5198035; doi:10.3390/ph9040060)
Supplement: Supplementary file 1 [file pharmaceuticals-09-00060-s001.pdf]

Supplementary Materials: pMPES: A Modular Peptide Expression System for the Delivery of Antimicrobial Peptides to the Site of Gastrointestinal Infections Using Probiotics

Kathryn Geldart, Brittany Forkus, Evelyn McChesney, Madeline McCue, and Yiannis N. Kaznessis

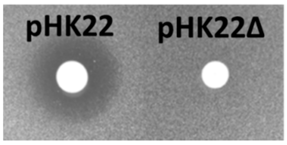

Figure S1. Abolishment of native Microcin V production by mutation of *cvaC*. Producer strain is *E. coli* MC1061 F′ and indicator strain is *E. coli* DH5α.

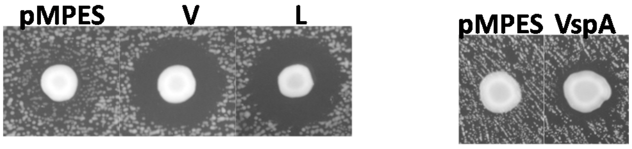

Figure S2. MccV, MccL, and EntA production from pMPES:V, L, and VspA in *E. coli* Nissle 1917. *E. coli* DH5α was used as the indicator strain for V and L and *E. faecium* 8E9 was used as the indicator strain for VspA. Note *E. coli* Nissle 1917 pMPES has native activity against *E. coli* DH5α but not *E. faecium* 8E9.

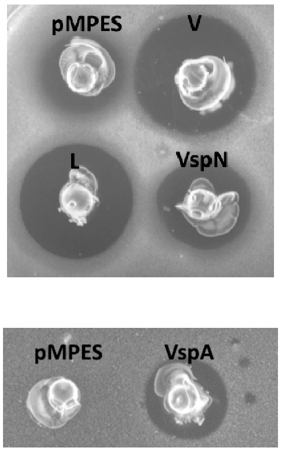

Figure S3. Alternative assay showing MccV, MccL, McnN and EntA production from pMPES:V, L, VspN, and VspA in *E. coli* Nissle 1917. *E. coli* DH5α was used as the indicator strain for V, L, and VspN and *E. faecium* 8E9 was used as the indicator strain for VspA. Note *E. coli* Nissle 1917 pMPES has native activity against *E. coli* DH5α but not *E. faecium* 8E9.

Table S1. Primers and DNA fragments used in this study.

Primer	Sequence
SDML	CTGCGCGCATGGTCTTC
SDM R	GATAAAAAGGAGATCATTAAAGAACTCTGACTCTA
SDM F	TAGAGTCAGAGTTCTTTAATGATCT CCTTTTTATC
SDML R	TTGAGATCTGTTGAG AGGGGTTTT
SDM_seq_F	CAGCAGCTGCTCCAAT
SDM_seq_R	ATTGCATTAGCTATATATGATTGTGT
Proteon_Assembler_F	GACAGCTTATCATCGATACTAGATAGATGACCTCGGG
Proteon_Assembler_R	CTGGCATTATGGTGGAAAGCTTCAGTTTAATTAAG<br>TGAGCTCACAGCTGTTTC
pHK22 HindIII Seq F	ATGTAGCACCTGAAGTCAGCCC
pHK22 HindIII Seq	GGTAATAGCGGTAAAGTGGCTAAACGG
MccV_SacI_F	TGCTAGAGCTCAAGTCAA GGCCGCATCG
MccV_PacI_R	CATAGTTAATTAATCTACAGTGAG CGGAAGGCC
AMP F	ATCCGGAGCTCTTAGCA
AMP R	TTGACTTAATTAATCGTAGGGGG
EntA_pHK22ΔP F	GAGAATTCAAGGAGGAAACAGCTGTGAGCTCTTAGCAGGAGGTGAACA<br>TGAAGCATTTGAAGATCCTGAGC
EntA_pHK22ΔP R	GGACATGTCCGTAGCGGCCGCACTGACCTAGGTTAGAACTGACTTTTATC<br>TCCATAGTCG
pMPES transition_F	GAGAATTCAAGGAGGAAACAGCTGTGAGCTCTTAGCAGGAGGTGAACA<br>TGAGAACTCTGACTCTAAATGAATTAGATTCT
pMPES transition_R	ACATGTCCGTAGCGGCCGCACTGACCTAGGCTCATGGGCCCGTCAACTG<br>TACGATCCTCTGAGCTTCG
MccV SeC F SpeI	CATAACTAGTATAGGAGGTTGTTTCGCCAGGAT
CvaB seq	ACAATCTGATCACAGGCGGT
PBS L_For	CTGATCATGCTCAAGGAGGAGTCAGATGAGAGAAATAACGTTAAATGA<br>AATG
PBS L_Rev	CTGTACGATCCTCTGAGCTTCGACCTCATTTGCCACGGTTGAA
PBS VA_For	CTGATCATGCTCAAGGAGGAGTCAGATGAGAACTCTGACTCTAAATGAA<br>TTAG
PBS VA_Rev	CTGTACGATCCTCTGAGCTTCGACCTTAGAACTGACTTTTATCTCCATAGT<br>CG
R_For	AAGGAGGAAACAGCTGTGAGCTCCTGATCATGCTCAAGGAGGAGTCAG
O_Rev	GGGCCCTCTGTAACTACTGTGGGAGCTCCTGTACGATCCTCTGAGCTTCG<br>ACC
O_For	CCACAGTAGTTACAGAGGGCCCCTGATCATGCTCAAGGAGGAGTCAG
YG_Rev	GCGGCCGCTATATGTTCTAGGCCTAGGGTCGCTATAGCTGCAGGGGCCC<br>CTGTACGATCCTCTGAGCTTCGACC

DNA Fragments	Sequence [1]
ProTeOn+	gacagcttatcatcgatactagatagatgacctcggggagcccgcctaatgagcgggctttttgcgcgccactctatc<br>attgactctatcattgatagagtacttaacataagcacctgtaggatcgtacaggtttagcgaagaaaatggtttgtta<br>tagtcgaataaacctcgagttatctcgagtgagatattgttgacgcaccaaggaggaagcttctatgatgagccgtct<br>ggataaaagcaaagtgattaatagcgcactggaactgctgaatgaagttggtattgaaggtctgaccacccgtaaa<br>ctggcccagaaactgggtgttgaacagccgaccctgtattggcatgtgaaaaataaacgtgcactgctggatgcact<br>ggccgttgaaattctggctcgccatcatgattatagcctgcctgcagcaggcgaaagctggcagagctttctgcgta<br>ataatgccatgagctttcgtcgtgccctgctgcgttatcgtgatggtgcaaaagaacatctgggcacccgtccggatg<br>aaaaacagtatgataccgttgaaacccagctgcgttttatgaccgaaaatggttttagcctgcgtgatggtctgtatgc<br>aattagcgcagttagccattttaccctgggtgccgttctggaacagcaggaacataccgcagcactgaccgatcgtc<br>ctccggcaccggatgaaaatctgcctccgctgctgcgtgaagcactgatgattatggattctgatgatggtgaacag<br>gcatttctgcatggtctggaaagcctgattcgtggttttgaagttcagctgaccgcactgctgcagattgttggtggtg<br>gtggtgcacgtacccagtatagcgaaagcatgggtgcccgtacacagtattctgaatctatgggtgctcgcacccag<br>tattcagaaagtatgggtgcaagaacacagtatagcgagtctatgggagcgcgtactcagtatagtgaatcaatgg<br>gaggtggtatgccgagcctggttgataattaccgcaagattaatattgccaataataaaagcaacaacgatctgacc<br>aaacgtgaaaaagaatgtctggcctgggcatgtgaaggtaaaagcagctgggatattagcaaaattctgggttgta<br>gcgaacgtaccgtgacctttcatctgaccaatgcccagatgaaactgaataccaccaatcgttgtcagagcattagca<br>aagcaattctgaccggtgccattgattgtccgtatttcaaaaactgagaattcaaggaggaaacagctgtgagctca<br>cttaattaaactgaagctttccaccataatgccag
MccV	gtcaaggccgcatcgaggaggaaacagct**atgagaactctgactctaaatgaattagattctgtttctggtggtGC**<br>**TTCAGGGCGTGATATTGCGATGGCTATAGGAACACTATCCGGGCAAT**<br>**TTGTTGCAGGAGGAATTGGAGCAGCTGCTGGGGGTGTGGCTGGAGG**<br>**TGCAATATATGACTATGCATCCACTCACAAACCTAATCCTGCAATGTC**<br>**TCCATCCGGTTTAGGGGGAACAATTAAGCAAAAACCCGAAGGGATA**<br>**CCTTCAGAAGCATGGAACTATGCTGCGGGAAGATTGTGTAATTGGAG**<br>**TCCAAATAATCTTAGTGATGTTTGTTTATAA**gataccaggaggaaactgct*ATGGA*<br>*TAGAAAAAGAACAAAATTAGAGTTGTTATTTGCATTTATAATAAATGCCACC*<br>*GCAATATATATTGCATTAGCTATATATGATTGTGTTTTTAGAGGAAAGGACTT*<br>*TTTATCCATGCATACATTTTGCTTCTCTGCATTAATGTCTGCAATATGTTACTT*<br>*TGTTGGTGATAATTATTATTCAATATCCGATAAGATAAAAAGGAGATCATAT*<br>*GAGAACTCTGACTCTAAATGA*aggtccatggtacgtacccatagataggcgccgttatcgactgggc<br>ctcatgggccttccgctcactg
MccL	atccggagctcttagcaggaggtgaac**atgagagaaataacgttaaatgaaatgaataatgtctctggtgctGG**<br>**TGATGTCAATTGGGTTGATGTCGGGAAAACTGTAGCAACAAACGGTG**<br>**CAGGAGTGATTGGCGGTGCATTCGGAGCTGGTCTGTGCGGCCCTGTT**<br>**TGTGCTGGTGCCTTCGCTGTTGGATCTTCTGCCGCTGTTGCTGCTTTG**<br>**TATGATGCAGCAGGAAATTCCAACTCAGCGAAACAAAAACCAGAAG**<br>**GACTACCTCCAGAAGCATGGAACTACGCTGAAGGTAGAATGTGTAAT**<br>**TGGAGTCCAAATAATCTTAGTGATGTTTGTTTATAA**actggaggaggtgcac*AT*<br>*GAAAACTTGGCAGGTTTTCTTCATCATCCTTCCGATCTCCATCATTATCTCTC*<br>*TGATTGTTAAACAGCTTAACAGCTCCAACCTTGTACAGAGCGTTGTAAGCGG*<br>*CATTGCTATCGCACTCATGATCTCCATCTTTTTCAACCGTGGCAAATGA*ttgacg<br>ggcccatgagcctaggtcagtgcggccgctacggacatgtccgta*gctagcgaaaaaaaaacccgcccctgacagggc*<br>*gggtttttttttaatttaacccccc*tacgattaattaagtcaa
Vsp:MccL	atccggagctcttagcaggaggtgaac**atgagaactctgactctaaatgaattagattctgtttctggtggtGGT**<br>**GATGTCAATTGGGTTGATGTCGGGAAAACTGTAGCAACAAACGGTG**<br>**CAGGAGTGATTGGCGGTGCATTCGGAGCTGGTCTGTGCGGCCCTGTT**<br>**TGTGCTGGTGCCTTCGCTGTTGGATCTTCTGCCGCTGTTGCTGCTTTG**<br>**TATGATGCAGCAGGAAATTCCAACTCAGCGAAACAAAAACCAGAAG**<br>**GACTACCTCCAGAAGCATGGAACTACGCTGAAGGTAGAATGTGTAAT**<br>**TGGAGTCCAAATAATCTTAGTGATGTTTGTTTATAA**actggaggaggtgcac*AT*<br>*GAAAACTTGGCAGGTTTTCTTCATCATCCTTCCGATCTCCATCATTATCTCTC*<br>*TGATTGTTAAACAGCTTAACAGCTCCAACCTTGTACAGAGCGTTGTAAGCGG*<br>*CATTGCTATCGCACTCATGATCTCCATCTTTTTCAACCGTGGCAAATGA*ttgacg<br>ggcccatgagcctaggtcagtgcggccgctacggacatgtccgta*gctagcgaaaaaaaaacccgcccctgacagggc*<br>*gggtttttttttaatttaacccccc*tacgattaattaagtcaa

McnN	gagaattcaaggaggaaacagctgtgagctcttagcaggaggtgaac**atgcgtgagctcgatcgtgaagaacttaactgcgtgggtggcgcgGGGGACCCACTCGCAGATCCTAACAGTCAGATCGTTCGCCAGATCATGTCCAATGCGGCCTGGGGGGCCGCCTTTGGTGCCAGAGGCGGTTTAGGGGGCATGGCGGTTGGTGCGGCTGGGGGCGTGACTCAAACAGTATTGCAGGGTGCTGCGGCGCATATGCCGGTCAACGTGCCGATTCCTAAGGTGCCGATGGGTCCTAGCTGGAACGGGTCCAAAGGCTAA**actggaggaggtgcac*ATGAGCTTTTTGAACTTTGCCTTTAGCCCCGTGTTTTTTTCCATCATGGCCTGCTATTTCATCGTATGGCGTAATAAACGCAATGAATTTGTTTGCAACCGCCTGCTGTCGATTATCATCATCTCATTTTTAATCTGCTTCATTTACCCATGGCTTAACTATAAAATTGAAGTGAAATACTACATCTTTGAACAGTTTTACTTGTTCTGCTTCCTGTCGAGCCTGGTTGCGGTAGTCATTAACCTGATCGTATACTTCATTCTGTACCGTCGCTGTATTTGA*ttgacgggcccatgagcctaggtcagtgcggccgctacggacatgtccgta*gctagcgaaaaaaaaacccgcccctgacagggcgggttttttttaatttaacccccc*tacgattaattaaactgaagctttccaccataatgcc
Vsp:McnN	gagaattcaaggaggaaacagctgtgagctcttagcaggaggtgaac**atgagaactctgactctaaatgaattagattctgtttctggtggtGGGGACCCACTCGCAGATCCTAACAGTCAGATCGTTCGCCAGATCATGTCCAATGCGGCCTGGGGGGCCGCCTTTGGTGCCAGAGGCGGTTTAGGGGGCATGGCGGTTGGTGCGGCTGGGGGCGTGACTCAAACAGTATTGCAGGGTGCTGCGGCGCATATGCCGGTCAACGTGCCGATTCCTAAGGTGCCGATGGGTCCTAGCTGGAACGGGTCCAAAGGCTAA**tgctaaggaggtgcta*ATGAGCTTTTTGAACTTTGCCTTTAGCCCCGTGTTTTTTTCCATCATGGCCTGCTATTTCATCGTATGGCGTAATAAACGCAATGAATTTGTTTGCAACCGCCTGCTGTCGATTATCATCATCTCATTTTTAATCTGCTTCATTTACCCATGGCTTAACTATAAAATTGAAGTGAAATACTACATCTTTGAACAGTTTTACTTGTTCTGCTTCCTGTCGAGCCTGGTTGCGGTAGTCATTAACCTGATCGTATACTTCATTCTGTACCGTCGCTGTATTTGA*ttgacgggcccatgagcctaggtcagtgcggccgctacggacatgtccgta*gctagcgaaaaaaaaacccgcccctgacagggcgggttttttttaatttaacccccc*tacgattaattaaactgaagctttccaccataatgcc
Lsp:EntA	atccggagctcttagcaggagg**tgaacatgagagaaataacgttaaatgaaatgaataatgtctctggtgct ACCACCCATAGCGGTAAGTATTACGGAAATGGAGTTTACTGTACCAAAAATAAATGCACCGTTGATTGGGCTAAAGCGACAACTTGTATCGCTGGTATGTCTATCGGCGGGTTCTTAGGGGGTGCCATTCCAGGCAAATGCTAA**tgctaaggaggtgcta*ATGAAGAAAAACGCCAAACAGATTGTTCACGAGTTATATAACGACATTAGTATCAGCAAAGACCCGAAGTATTCGGACATCCTTGAGGTACTGCAAAAAGTGTATCTGAAACTGGAAAAACAGAAATATGAGTTAGATCCAGGACCATTGATCAATCGTCTGGTAAACTACCTGTACTTCACCGCCTATACCAACAAGATTCGTTTCACCGAATATCAAGAAGAGTTAATCCGCAATCTGTCTGAGATTGGACGCACAGCAGGAATCAACGGACTGTACCGTGCCGACTATGGAGATAAAAGTCAGTTCTAA* ttgacgggccc atgagcctagg tcagtgcggccgc tacggacatgt ccgta *gctagcgaaaaaaaaacccgcccctgacagggcgggttttttttaatttaacccccc*tacgattaattaagtcaa
Vsp:EntA (for pMPES)	gagaattcaaggaggaaacagctgtgagctcttagcaggaggtgaac**atgagaactctgactctaaatgaattagattctgtttctggtggtACCACTCATAGCGGTAAGTATTACGGAAATGGAGTTTACTGTACCAAAAATAAATGCACCGTTGATTGGGCTAAAGCGACAACTTGTATCGCTGGTATGTCTATCGGCGGGTTCTTAGGGGGTGCCATTCCAGGCAAATGCTAA**tgctaaggaggtgcta*ATGAAGAAAAACGCCAAACAGATTGTTCACGAGTTATATAACGACATTAGTATCAGCAAAGACCCGAAGTATTCGGACATCCTTGAGGTACTGCAAAAAGTGTATCTGAAACTGGAAAAACAGAAATATGAGTTAGATCCAGGACCATTGATCAATCGTCTGGTAAACTACCTGTACTTCACCGCCTATACCAACAAGATTCGTTTCACCGAATATCAAGAAGAGTTAATCCGCAATCTGTCTGAGATTGGACGCACAGCAGGAATCAACGGACTGTACCGTGCCGACTATGGAGATAAAAGTCAGTTCTAA*ttgacgggcccatgagcctaggtcagtgcggccgctacggacatgtccgta*gctagcgaaaaaaaaacccgcccctgacagggcgggttttttttaatttaacccccc*tacgattaattaaactgaagctttccaccataatgcc

EntA	gagaattcaaggaggaaacagctgtgagctcctgatcatgctcaaggaggagtcag**atgaagcatttgaagatcctgagcattaaggagACCCAACTGATATACGGAGGTACCACTCATAGCGGTAAGTATTACGGAAATGGAGTTTACTGTACCAAAAATAAATGCACCGTTGATTGGGCTAAAGCGACAACTTGTATCGCTGGTATGTCTATCGGCGGGTTCTTAGGGGGTGCCATTCCAGGCAAATGCTAA**tcgtaggaggacgtac*ATGAAGAAAAACGCCAAACAGATTGTTCACGAGTTATATAACGACATTAGTATCAGCAAAGACCCGAAGTATTCGGACATCCTTGAGGTACTGCAAAAAGTGTATCTGAAACTGGAAAAACAGAAATATGAGTTAGATCCAGGACCATTGATCAATCGTCTGGTAAACTACCTGTACTTCACCGCCTATACCAACAAGATTCGTTTCACCGAATATCAAGAAGAGTTAATCCGCAATCTGTCTGAGATTGGACGCACAGCAGGAATCAACGGACTGTACCGTGCCGACTATGGAGATAAAAGTCAGTTCTAA*ggtcgaagctcagaggatcgtacaggagctcccacagtagttacagagggcccctgcagctatagcgaccctaggcctagaacatatagcggccgcccatcacactgtgagcacatgtggacacttcaccacgtatctgg*gctagcgaaaaaaaaacccgcccctgacagggcgggttttttttttaatttaacccccc*tacgattaattaaactgaagctttccaccataatgcc
pMPES MCS	gagaattcaaggaggaaacagctgtgagctcccacagtagttacagagggcccctgcagctatagcgaccctaggcctagaacatatagcggccgcccatcacactgtgagcacatgtggacacttcaccacgtatctgg*gctagcgaaaaaaaaacccgcccctgacagggcgggttttttttttaatttaacccccc*tacgattaattaaactgaagctttccaccataatgcc
Vsp:EntA (for pMPESb)	aaggaggaaacagctgtgagctcctgatcatgctcaaggaggagtcag**atgagaactctgactctaaatgaattagattctgtttctggtggtACCACTCATAGCGGTAAGTATTACGGAAATGGAGTTTACTGTACCAAAAATAAATGCACCGTTGATTGGGCTAAAGCGACAACTTGTATCGCTGGTATGTCTATCGGCGGGTTCTTAGGGGGTGCCATTCCAGGCAAATGCTAA**ggtcgaagctcagaggatcgtacaggagctcccacagtagttacagagggccc
Vsp:HirJM79	aaggaggaaacagctgtgagctcctgatcatgctcaaggaggagtcag**atgagaactctgactctaaatgaattagattctgtttctggtggtGCGACATACTATGGAAATGGATTGTATTGCAATAAGGAGAAGTGTTGGGTCGATTGGAATCAAGCTAAAGGAGAGATCGGAAAGATAATAGTGAACGGTTGGGTGAATCATGGTCCTTGGGCTCCCAGACGCTAA**ggtcgaagctcagaggatcgtacaggagctcccacagtagttacagagggccc
Vsp:EntP	ctgatcatgctcaaggaggagtcag**atgagaactctgactctaaatgaattagattctgtttctggtggtGCAACTCGCTCATACGGGAATGGGGTGTATTGTAACAATTCTAAATGTTGGGTGAACTGGGGTGAAGCGAAAGAAAACATCGCTGGTATCGTCATCTCTGGCTGGGCCTCAGGACTTGCAGGAATGGGTCATTAA**ggtcgaagctcagaggatcgtacaggagctcccacagtagttacagagggccc
Vsp:EntB	aaggaggaaacagctgtgagctcctgatcatgctcaaggaggagtcag**atgagaactctgactctaaatgaattagattctgtttctggtggtGAAAACGACCACAGAATGCCCAACGAGTTGAATCGCCCTAACAATCTTAGCAAAGGGGGAGCCAAATGCGGCGCGGCGATTGCAGGTGGACTTTTCGGGATACCGAAAGGACCGCTGGCTTGGGCCGCTGGATTAGCGAATGTTTACTCAAAATGTAACTAA**ggtcgaagctcagaggatcgtacaggagctcccacagtagttacagagggccc
pMPES RBS	gagctcttagcaggaggtgaac
pMPESb RBS	gagctcctgatcatgctcaaggaggagtcag

[1]: The following key is used to label components of AMP DNA fragments. Ribosomal Binding Sites: lowercased, underlined; Signal peptide: lowercased, bold; Mature peptide: uppercased, bold; Immunity Gene: uppercased, italicized; Terminator: lowercased, italicized, underlined.
